# Immunogenetic investigation of WAS patients revealing impaired IL-6/STAT3 signaling in T cells

**DOI:** 10.3389/fimmu.2025.1602942

**Published:** 2025-09-16

**Authors:** Firas Bouzakoura, Najla Mekki, Monia Ben Khaled, Ines Maaloul, Aicha Ben Taieb, Amal Zammeli, Meriem Ben-Ali, Mariem Tira, Ansem Ben-Hammadi, Thouraya Kamoun, Monia Ouederni, Mohamed-Ridha Barbouche, Imen Ben-Mustapha

**Affiliations:** ^1^ Laboratory of Transmission, Control and Immunobiology of Infections, LR16IPT02, Institut Pasteur de Tunis, University of Tunis El Manar, Tunis, Tunisia; ^2^ Faculty of Medicine of Tunis, University of Tunis El Manar, Tunis, Tunisia; ^3^ Department of Pediatrics, National Center of Bone Marrow Transplantation, Tunis, Tunisia; ^4^ Department of Pediatrics, Hedi Chaker University Hospital, Sfax, Tunisia; ^5^ Department of Microbiology, Immunology and Infectious Diseases, College of Medicine and Health Sciences, Arabian Gulf University, Manama, Bahrain

**Keywords:** WAS, HIES, DOCK8, STAT3, IL-6

## Abstract

Wiskott-Aldrich syndrome (WAS) is an inborn error of immunity caused by loss-of-function mutations in the *WAS* gene, which encodes WASp, a key regulator of cytoskeletal remodeling. In addition to microthrombocytopenia, affected individuals often present with recurrent infections, eczema, eosinophilia and elevated IgE levels, suggesting a potential pathophysiological overlap with STAT3 hyper-IgE syndrome (HIES). Given these shared features, we investigated the immunogenetic characteristics of three WAS patients and explored the IL-6/STAT3 pathway as a potential underlying mechanism. Flow cytometry revealed absent WASp expression in P1 and P3, while P2 showed reduced level. Genetic analysis identified three hemizygous mutations: A56V substitution within the WH1 domain in P1, and two splice site mutations, c.360 + 1G>T and c.734 + 1G>C, in P2 and P3, respectively. Interestingly, all WAS patients showed impaired STAT3 phosphorylation in T cells following IL-6 stimulation and SOCS3 induction was markedly decreased. These results further suggest a potential link between WASp and STAT3. Considering the interaction of WASp with DOCK8-WIP in lymphocytes and the critical role of DOCK8 in regulating STAT3 phosphorylation following IL-6 stimulation, we analyzed DOCK8 expression in lymphoblastoid cell lines. We demonstrated normal DOCK8 levels suggesting that STAT3 signaling defect is due to the absence of WASp rather than DOCK8 loss. Our results demonstrate the impairment of T cell IL-6/STAT3 pathway in WAS patients which could underlie, in part, the overlapping phenotype with HIES patients.

## 1 Introduction

Wiskott-Aldrich Syndrome (WAS) is a combined syndromic inborn error of immunity (IEI) caused by loss-of-function mutations in the *WAS* gene, located on the X chromosome (1). This gene encodes the Wiskott-Aldrich Syndrome protein (WASp), which is exclusively expressed in non-erythroid hematopoietic cells and plays a central role in cytoskeletal remodeling. Thrombocytopenia is the hallmark manifestation of this disease. Patients with complete clinical presentation, also experience recurrent infections, eczema, eosinophilia and elevated IgE levels (2, 3). These symptoms are a hallmark of Hyper-IgE Syndromes (HIES), particularly those caused by STAT3 and DOCK8 deficiencies (4).

Autosomal dominant Hyper-IgE Syndrome (AD-HIES) also known as Buckley syndrome is characterized by eczema, recurrent staphylococcal skin abscesses, pneumonias, and mucocutaneous candidiasis. Additionally, malformative and connective tissue abnormalities, such as retained primary teeth, joint hyperextensibility and facial dysmorphism, are distinctive manifestations of this condition (5). While the clinical features of AD-HIES have been well-documented for years, it was not until 2007 that heterozygous loss-of-function (LOF) mutations in the *STAT3* gene were identified as the root cause of this disorder (6, 7). *STAT3* gene encodes a crucial transcription factor involved in cytokine signaling pathways downstream multiple cytokines including IL-6. Upon IL-6 binding, the IL-6 receptor complex (IL-6R and gp130) undergoes dimerization, triggering JAK kinase activation, phosphorylation of gp130 and subsequent recruitment of STAT3. Once phosphorylated at Tyr705, STAT3 forms dimers and translocates to the nucleus to regulate the expression of target genes (8). IL6/STAT3 signaling plays a pivotal role in promoting the differentiation of Th17 cells, T follicular helper (Tfh) cells and RORγt^+^ regulatory T (Treg) cells, while concurrently limiting Th2 polarization through SOCS3-mediated inhibition of the IL-2/STAT5 pathway (9, 10).

Several patients with autosomal recessive Hyper-IgE Syndrome (AR-HIES), carrying mutations in the *DOCK8* gene were subsequently described in 2009 (11, 12). Clinically, patients with DOCK8 deficiency experience recurrent infections, including bacterial skin abscesses mainly caused by *Staphylococcus*, mucocutaneous fungal infections, recurrent respiratory infections as well as eczema. They also present with elevated IgE levels and eosinophilia and have a heightened risk of developing malignancies (13). DOCK8 is a guanine nucleotide exchange factor highly expressed in leukocytes, particularly lymphocytes (12, 14). It plays a crucial role in activating Rho family small GTPases, including CDC42 and RAC, thereby integrating membrane-derived signals to regulate pathways essential for actin cytoskeleton dynamics (15). This process also involves the DOCK8-WIP-WASp complex following TCR activation (16). Additionally, DOCK8 mediates IL-6/STAT3 activation in T cells and this pathway is known to be impaired in DOCK8 deficient patients (17, 18). Furthermore, DOCK8forms a complex with WASp to control IL-10/STAT3 signaling in macrophages (19).

In this study, we investigated the immunogenetic characteristics of three WASp deficient patients and explored the IL-6/STAT3 signaling pathway as a potential underlying mechanism for the overlap between WAS and HIES.

## 2 Methods

### 2.1 Flow cytometric experiments

#### 2.1.1 Phenotypic analysis

For lymphocyte phenotyping, standard flow cytometric methods were used. Cells were incubated with various combinations of labeled monoclonal antibodies (anti-CD3, anti-CD4, anti-CD8, anti-CD19, anti-CD16/56, anti-CD45RA, anti-CXCR5, anti-IgD, anti-CD27) (BD Biosciences) for 30 minutes in the dark at 4 °C. Subsequently, the cells were washed once with Phosphate Buffered Saline (PBS) 0.3% Bovine Serum Albumin (BSA) by centrifugation at 1500 rpm for 8 min at +4 °C, fixed with 300 µl of PBS containing 1% paraformaldehyde and analyzed by flow cytometry.

#### 2.1.2 Intracellular WASp expression analysis

Peripheral blood mononuclear cells (PBMCs) were isolated from heparinized venous blood by Ficoll-Hypaque density gradient centrifugation. The cells were then fixed, permeabilized and stained with either anti-WASp or isotype control antibody, followed by incubation with a secondary antibody. After surface staining with anti-CD3 antibody, the samples were analyzed by flow cytometry.

The detailed protocol is provided in the **Supplementary Materials**.

#### 2.1.3 STAT3 phosphorylation analysis

STAT3 phosphorylation was analyzed as previously described (20). Briefly, PBMCs were treated with IL-6 (20 ng/mL; R&D Systems, Inc., Minneapolis, MN) for 15 minutes at 37 °C followed by fixation at 37 °C with Cytofix buffer (BD Biosciences) for 10 minutes. Subsequently, the cells were permeabilized on ice with Perm Buffer III for 30 minutes (BD Biosciences) before being labeled with an anti-phospho-STAT3 (pY705) antibody (BD Biosciences) for 1 hour at 4 °C. After a wash, cells were incubated with anti-CD3 antibody for 20 minutes and resuspended in PBS for flow cytometry analysis.

All flow cytometric analyses were recorded on a BD FACS CantoTM II (BD Life Sciences) and the data were processed with Diva (BD Bioscience, San Jose, CA, USA) and FlowJo softwares.

### 2.2 Proliferation assays

PBMCs were cultured in RPMI supplemented with 7%AB serum and incubated with phytohemagglutinin (PHA) (5μg/ml) or anti-CD3 for 3 days and with specific antigens (tuberculin purified protein derivative (PPD; 20μg/ml)) for 5 days at 37 °C/5% CO2. *In vitro* proliferation of lymphocytes was measured by assessing the 3H-thymidine incorporation.

### 2.3 Genetic analysis

Genomic DNA was isolated from EDTA-peripheral blood samples using the QIAmp DNA Mini Kit (Qiagen, Hilden, Germany).

The 12 exons of *WAS* gene containing the flanking splice sites were analyzed by Sanger sequencing. The primers used for amplification, along with the different conditions for amplifying each gene segment, are detailed in **Supplementary Materials**. Amplified PCR products were purified with the EXO-SAP clean-up procedure (Amersham Biosciences) and sequenced using Big Dye Terminator (V3.1) chemistry. Sequence files and chromatograms were analyzed with GENALYS software (CNG, France).

### 2.4 Real-time PCR analysis of *SOCS3* gene expression

*Suppressor of Cytokine Signaling 3 (SOCS3)* mRNA gene expression was quantified by real-time PCR using SYBR Green in PBMCs. Cells were stimulated for 2 hours with recombinant human IL-6 (100 ng/mL, R&D Systems) prior to RNA extraction and cDNA synthesis. PCR reactions were performed on a QuantStudio 3 thermocycler. β-actin was used as the housekeeping gene for normalization. Relative gene expression was analyzed using the 2^–ΔCT^ method.

### 2.5 Western blot analysis of DOCK8 expression

Western blot for DOCK8 protein expression was performed on Epstein-Barr virus-transformed lymphoblastoid cell lines (B-LCLs) derived from a WAS patient and a healthy control. Briefly, proteins were extracted and quantified using Bicinchoninic Acid (BCA) Protein Assay, then separated by electrophoresis and transferred on to a PVDF membrane. The membranes were probed with either a monoclonal anti-DOCK8 or anti-β-actin antibody, followed by an HRP-conjugated secondary antibody. Protein detection was carried out with enhanced chemiluminescence (ECL).

The detailed protocol is provided in the **Supplementary Materials**.

## 3 Results

### 3.1 Case description and preliminary immunological investigation

Three patients belonging to 3 unrelated families and presenting with a clinical presentation suggestive of WAS, were investigated.

P1 was born at 35 weeks of gestation to third-degree consanguineous parents, with no notable family history. During the neonatal period, thrombocytopenia and anemia were identified, along with a positive anti-platelet antibodies. At seven months of age, he developed eczema and persistent thrombocytopenia without bleeding or recurrent infections. Repeated blood cell counts consistently showed a low mean platelet volume (MPV) (<5 fL) and a peripheral blood smear confirmed the presence of small platelets, raising suspicion of WAS and prompting referral for specialized care. No neurological, pleuropulmonary or cardiovascular impairments were observed, and there were no signs of cutaneous or mucosal bleeding, tumor syndrome or palpable lymphadenopathy. Bone marrow smear analysis revealed a polymorphic marrow with well-represented cell lineages. The granulocytic lineage was present at all maturation stages with a distinct eosinophilic component. Immunological evaluation showed eosinophilia, basophilia and hypogammaglobulinemia with low IgG and IgM. Serum IgE levels were not assessed. Lymphocyte phenotyping revealed decreased total memory and non-switched memory B-cells and elevated NK-cell levels. Proliferation in response to anti-CD3 stimulation was impaired. The patient was managed with regular intravenous immunoglobulin (IVIG) infusions and antibiotic prophylaxis. At the age of 8 years, a trauma induced severe epistaxis episode requiring platelet transfusion and immunoglobulin therapy. His follow-up at age 11 years showed a favorable outcome with no severe infections and only mild bleeding episodes not requiring specific treatment. Currently, hematopoietic stem cell transplantation (HSCT) has not been considered due to the absence of a geno-identical donor and the relatively mild severity of his clinical presentation.

P2, a full-term male infant born to non-consanguineous parents, presented at 7 days of age with omphalitis. Initial evaluation revealed atopic dermatitis localized to the forehead with dry skin, thrombocytopenia with reduced MPV but no bleeding and regenerative hemolytic anemia with a negative direct Coombs test. At 2 months, he developed localized BCGitis. Peripheral blood smear confirmed the presence of small platelets and bone marrow analysis was unremarkable. Immunological workup showed slightly increased IgE, basophilia, lymphopenia and persistent thrombocytopenia. Lymphocyte immunophenotyping revealed reduced CD3+, CD4+, CD8+, naïve CD4+ T cells and CXCR5+ memory helper T cells with increased NK cells. Proliferation responses to mitogens and specific antigens were normal. The patient had persistent thrombocytopenia with recurrent severe bleeding (rectal bleeding, hematuria, and cutaneous hemorrhages) despite regular IVIG therapy and platelet transfusions. At the age of five years, he developed facial herpes zoster (resolved with acyclovir) followed by recurrent herpes labialis with gingivitis starting at age 10. On the latest follow-up, his skin remained dry with eczematous lesions. Given the high risk of life-threatening bleeding and a disease severity score of 4 (thrombocytopenia, eczema, immunodeficiency and autoimmunity), he is currently being evaluated for a haploidentical bone marrow transplant from his father. Treatment with eltrombopag is also under consideration to reduce the bleeding risk.

P3 was born to non-consanguineous parents without an apparent family history. Clinical manifestations started at 48 hours of life and were marked by bruising and petechial purpura. A complete blood cell count revealed isolated thrombocytopenia. He experienced multiple hospitalizations, starting at 3 months for persistent thrombocytopenia requiring platelet transfusion. At 7 months, he was admitted again for thrombocytopenia, poorly tolerated anemia, and generalized eczema. By 16 months, he developed autoimmune hemolytic anemia (AIHA) with a positive IgG direct Coombs test, with ongoing thrombocytopenia. Laboratory investigations revealed small platelets with a MPV of 4.9 fL. Bone marrow biopsy results were consistent with peripheral thrombocytopenia. Antinuclear antibody testing was positive with a speckled pattern. Immunoglobulin profiling revealed reduced IgM, increased IgA and markedly elevated IgE levels. Lymphocyte immunophenotyping showed decreased CD8+ T cells, naïve CD4+ T cells, total memory and non-switched memory B-cells as well as CXCR5+ memory helper T cells. NK cells were elevated. Proliferation responses were impaired to anti-CD3 stimulation. The patient was managed with antibiotic prophylaxis, monthly IVIG infusions, corticosteroid therapy for AIHA and topical corticosteroids for eczema. At 2 years and 7 months, the patient developed candidemia, which was successfully treated with systemic antifungal therapy over one month. At 3 years of follow-up, the patient remained free of bleeding episodes. Given the severity of his clinical phenotype and the absence of a geno-identical donor, a haploidentical hematopoietic stem cell transplant is currently under consideration for this patient.

Based on clinical presentation and initial investigations, P1 was diagnosed with X-linked thrombocytopenia (XLT) with a severity score of 2, while P2 and P3 exhibited classic WAS with a score of 4 and 5 respectively.

The detailed immunological phenotype is summarized in **Table 1**.

**Table 1:** Clinical and immunogenetic features of WAS patients.

Patients	P1	P2	P3
**Age at diagnosis**	**18 months**	**2 months**	**6 months**
*Clinical symptoms*			
** Eczema**	**+**	**+**	**+**
** Infections**	**-**	Local BCGitis, omphalitis, facial herpes zoster, recurrent herpes labialis with gingivitis	Severe candidiasis
** Autoimmunity**	**-**	+	+
** Additional features**	**-**	**-**	Hypotrophy
*Immnoglobulin Levels*			
IgM *(g/L)*	**0.22 ↓** (0.4-2.29)	0.2 (0.2-1.2)	**0.27↓** (0.48-2.28)
IgA *(gLl)*	0.37 (0.13-1.02)	0.37 (0.04-0.78)	**1.93 ↑** (0.13-0.82)
IgG *(g/L)*	6.08 * (3.49-11.39)	7.58 (1.39-9.34)	7.11*(4.1-10.81)
IgE (UI/mL)	–	**15.1 ↑** (<7)	**2500 ↑** (<70)
*Hemogram*			
Leucocyte (cells/mL)	21 200	7600	17 800
Neutrophil (cells/mL)	5000	1900	9400
Lymphocyte (cells/mL)	10330	4160	5200
Basophil (cells/mL)	**400 ↑**	**140 ↑**	50
Eosinophil (cells/mL)	**3900 ↑**	200	460
Hemoglobin (g/dL)	**9.5 ↓**	**9.6 ↓**	**7.7 ↓**
Platelets (cells/mL)	**4200 ↓**	**4000 ↓**	**29000 ↓**
Small platelets	**+**	**+**	**+**
*Lymphocyte subsets* (% / count *10^-3^/μL)			
CD3+	**55.5 ↓** (56-75)/5.7 (2.1-6.2)	**43.7 ↓ (**51-77)/**1.8 ↓** (2.5-5.5)	64 (51–77)/3.3 (2.5-5.6)
CD4+	40.5 (28-47)/**4.1↑** (1.3-3.4)	**34 ↓** (35-56)/**1.41 ↓** (1.6-4)	55 (35–56)/2.8 (1.8-4)
CD8+	18 (16-30)/1.8 (0.62-2)	12.2 (12-23)/**0.5 ↓** (0.56-1.7)	**6↓ (**12–23)/**0.312 ↓** (0.56-1.7)
CD19^+^	20 (14-33)/2.6 (0.72-2.6)	19 (11-41)/ 0.79 (0.3-2)	13.5 (11–41)/0.612 (0.3-2)
CD16^+^/CD56^+^	**18 ↑** (4-17)/**1.85 ↑** (0.18-0.92)	**31 ↑** (3-14)/**1.28 ↑** (0.17/1.1)	**17.5↑** (3-14)**/0.91↑** (0.17-0.83)
CD19^+^/CD27^+^	**6.1 ↓** (9.0–35.0)	**-**	**3.1- ↓** (3.5–12.2)
CD19^+^/CD27^+^/IgD^-^	6 (4.4–20.5)	**-**	2 (0.6–3.7)
CD19^+^/CD27+/IgD^+^	**0.9 ↓** (3.0–21.1)	**-**	**1 ↓** (2.4–9.9)
CD4^+^/CD45RA^+^	**-**	**16.6 ↓** (53-86)	**32 ↓** (46-77)
CXCR5^+^ memory helper T cells **§**	14.6 (7–47)	**3.3 ↓ (**7 - 85)	**5.5 ↓** (7–47)
*T-Cell Proliferation (cpm/IS)*			
PHA	83616/38	31631/66	62312/70
Anti-CD3	**6990/2 ↓**	15164/31	**1906/1 ↓**
Tuberculin	**1962/0.7 ↓**	7492/32	**460/0 ↓**
*WASp expression*			
(%)	**0%**	**32%↓**	**0%**
*Genetic analysis*			
*WAS* mutation	**c.167C>T**	**c.360+1G>T**	**c.734+1G>C**

* Patients treated with IVIG, **§**: % of CD4^+^CD45RA^-^ T lymphocytes, CD19^+^/CD27^+^: total memory B cells, CD19^+^/CD27^+^IgD^-^: switched memory B-cells, CD19+/CD27+IgD+: non-switched memory B cells. Values shown in bold correspond to abnormal results. The symbols ↑ and ↓ are used to denote, respectively, increased and decreased values.

### 3.2 Diagnostic assessment and IL-6/STAT3 pathway investigation

**Figure 1:**
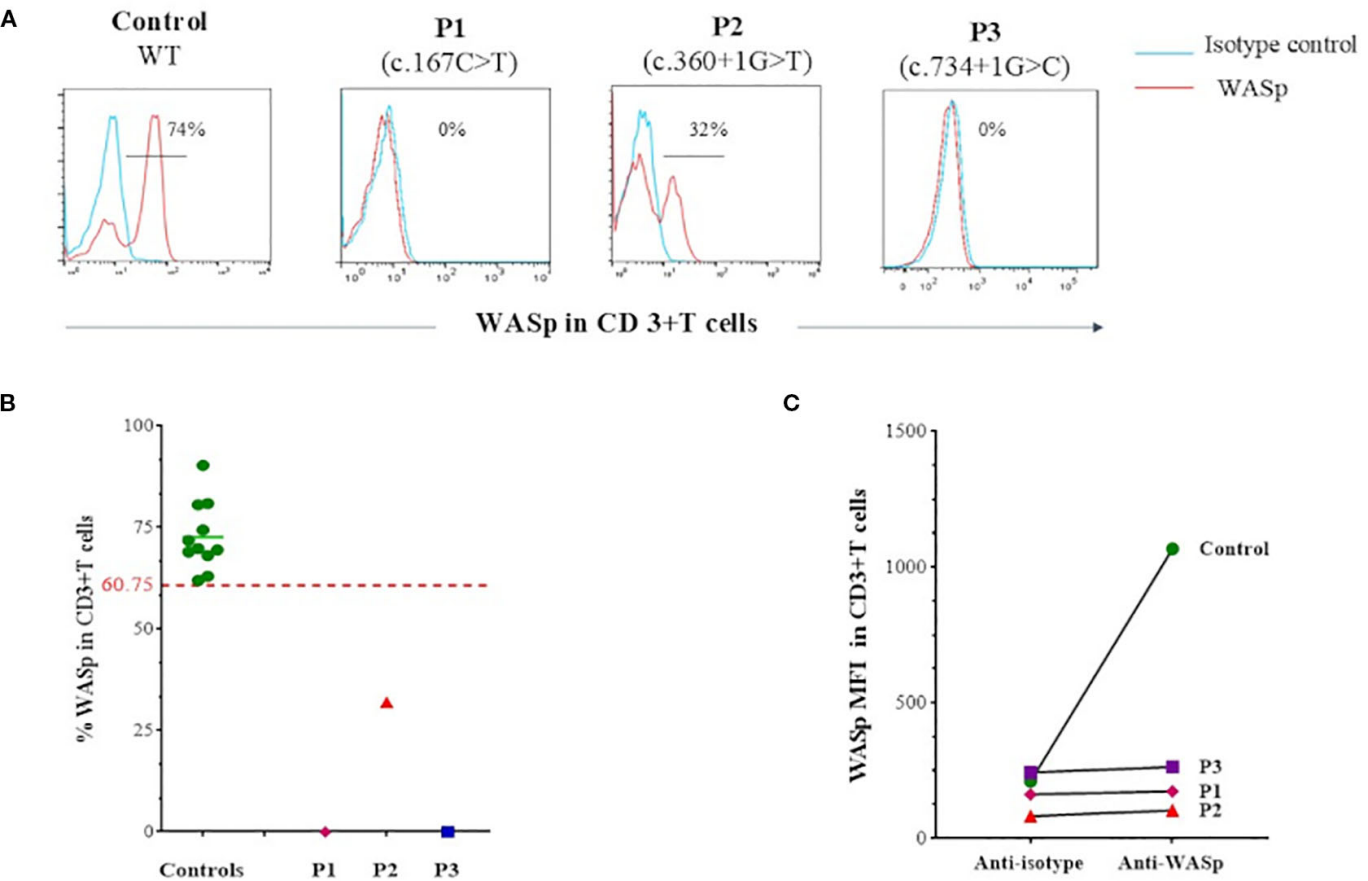
Flow cytometry analysis of intracellular WASp expression. **(A)** Flow cytometry histograms showing intracellular WASp expression in CD3^+^ T cells from patients compared to healthy control. In blue: isotype-matched IgG1 control; in red: anti-WASp monoclonal antibody staining. **(B)** Percentage of WASp expression in CD3^+^ T cells, based on isotype control, as assessed by flow cytometry in patients compared to a group of healthy controls. Threshold value was determined according to the 5th percentile (95% confidence interval) in control groups. **(C)** Mean fluorescence intensity (MFI) of intracellular WASp expression in CD3^+^ T cells as assessed by flow cytometry in patients compared to a control.

Given the presence of eczema, small platelets and thrombocytopenia, WAS fell suspected. Flow cytometry analysis of intracellular WASp expression confirmed the diagnosis, revealing a complete absence of WASp in P1 and P3, while P2 exhibited reduced WASp level compared to healthy controls (**Figure 1**).

**Figure 2:**
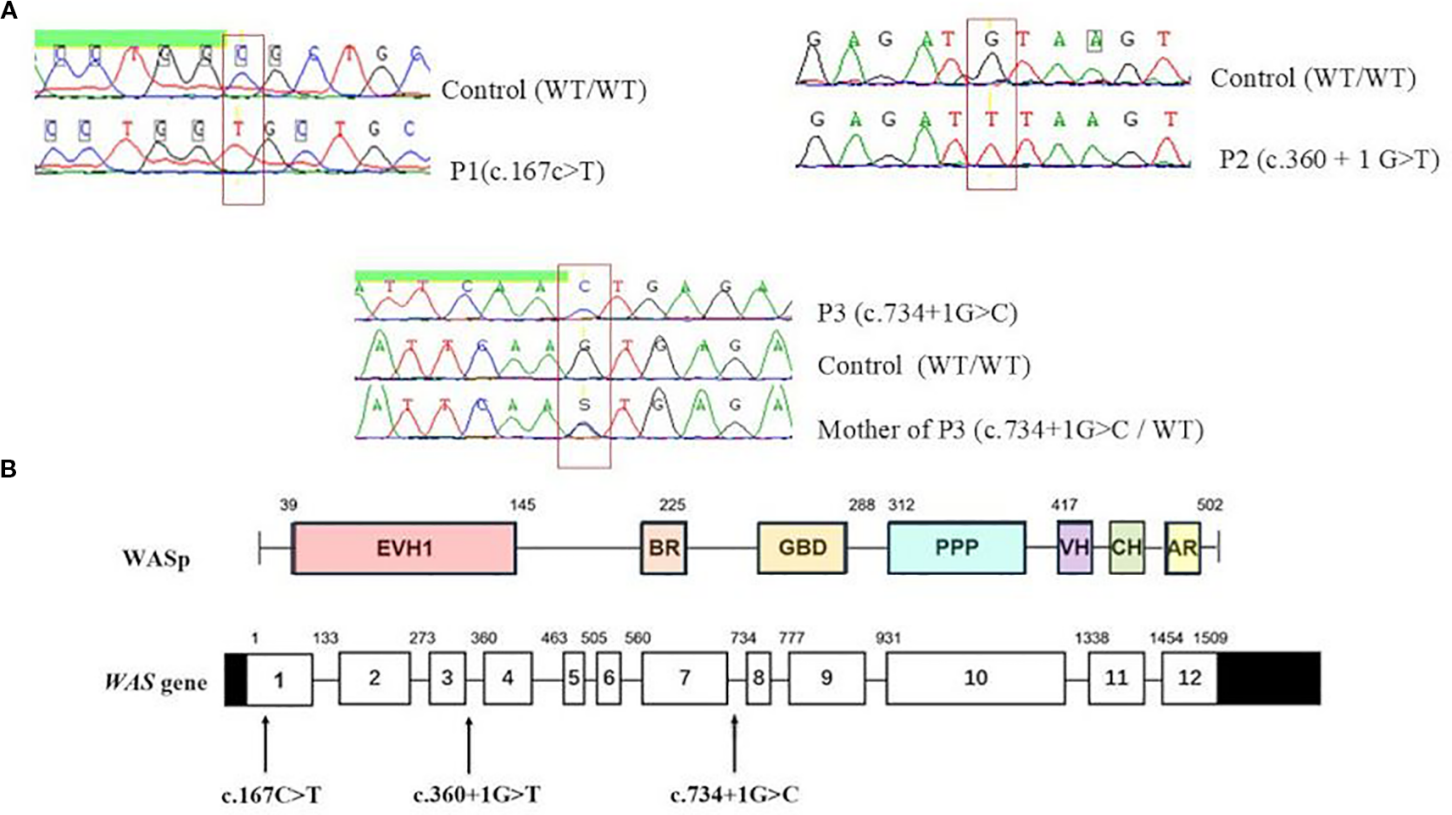
Molecular analysis of *WAS* gene in patients. **(A)** Sanger sequencing of the *WAS* gene identified three distinct hemizygous mutations. **(B)** Distribution of identified variants on *WAS* gene. EVH1, Ens/VASP homology 1 domain; BR, basic region; GBD, GTPase bending domain; PPPP, proline-rich region; VH, verprolin homology; CH, cofilin homology; AR, acidic region.

Sanger sequencing of the WAS gene revealed three distinct hemizygous mutations (**Figure 2A**). P1 carried a missense mutation (c.167C>T) resulting in a p.A56V substitution within the WH1 domain (**Figure 2B**), predicted as deleterious by MutationTaster software. In P2, a previously reported splice-donor-site mutation in intron 3 (c.360 + 1G>T) was identified, predicted to disrupt normal splicing. P3 showed a described hemizygous splice-site substitution in intron 7 (c.734 + 1G>C), which was also predicted to disrupt normal splicing. Genetic testing of P3’s mother confirmed her heterozygous carrier status (**Figure 2A**).

**Figure 3:**
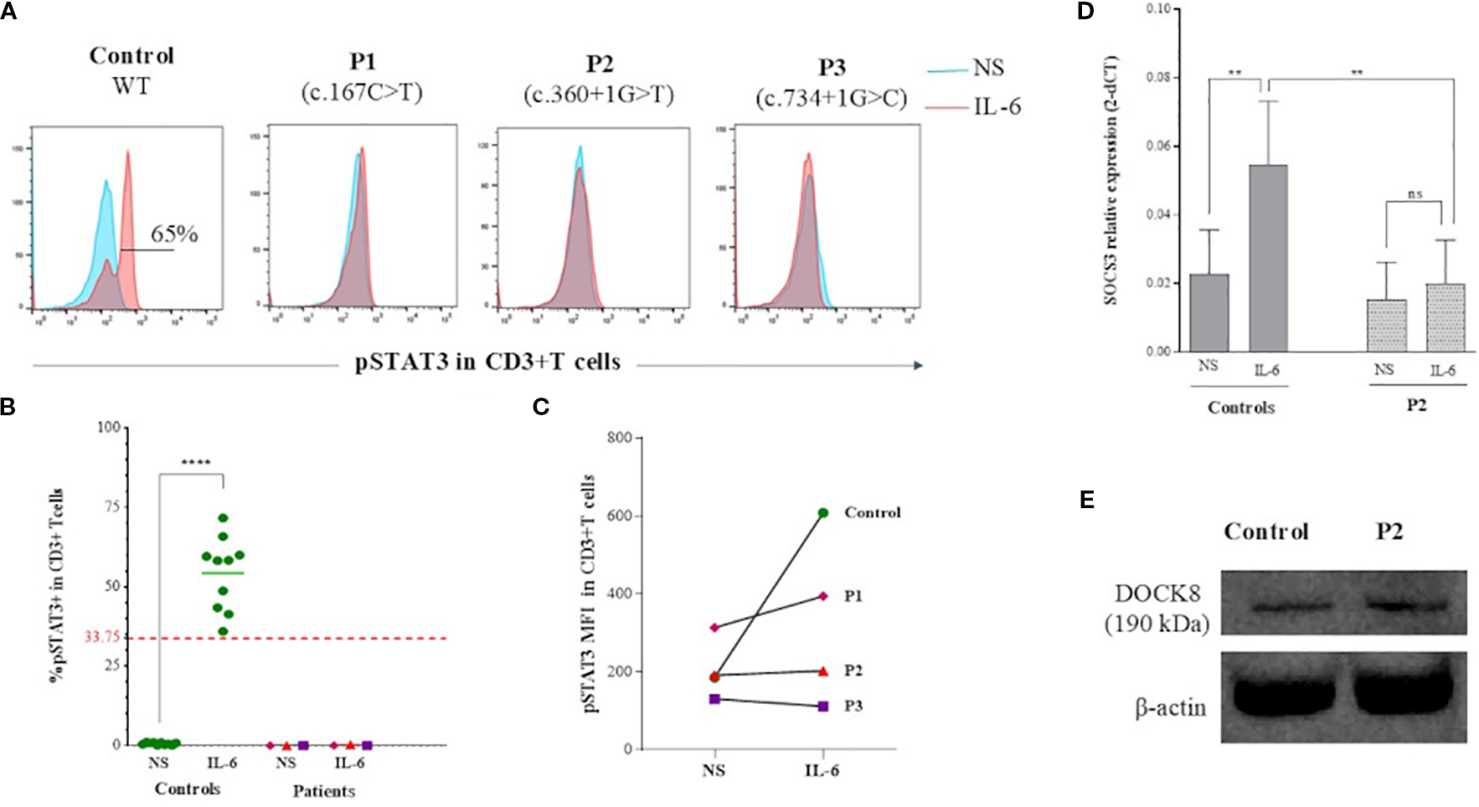
IL-6/STAT3 pathway analysis in WASp deficient T cells. **(A)** Flow cytometry histograms showing phosphorylation of STAT3 at Tyr705 (pY705-STAT3) in CD3^+^ T cells from WAS patients and a healthy control. In blue: unstimulated PBMCs; in red: PBMCs stimulated with IL-6 **(B)** Percentage of pY705-STAT3 expression in CD3^+^T cells as assessed by flow cytometry in patients compared to a group of healthy controls. Threshold value was determined according to the 5th percentile (95% confidence interval) in control groups. Statistical significance was assessed using an unpaired t-test. **(C)** MFI of pY705-STAT3 expression in CD3+T cells assessed by flow cytometry in patients compared to control. **(D)** SOCS3 mRNA expression measured by quantitative RT-PCR in IL-6–stimulated PBMCs from P2 and three healthy controls. Data are displayed as 2^–ΔCT^ after normalization relative to β-actin expression. Error bars indicate standard deviation. Statistical significance was assessed using an unpaired t-test. **(E)** Immunoblot analysis of DOCK8 expression in B-LCLs derived from P2 and a healthy control, showing comparable expression levels.

Then, in order to investigate the underlying mechanism for the shared features between WAS and HIES, we studied the IL-6/STAT3 signaling pathway. All WAS deficient patients showed a complete absence of STAT3 phosphorylation in T cells following IL-6 stimulation whereas, healthy controls showed normal phosphorylation (**Figures 3A–C**). The induction of *SOCS3*, a STAT3 target gene (21), in P2’s PBMCs stimulated with IL-6 was significantly decreased compared to three healthy controls (*p value:0.0039*) (**Figure 3D**).

To determine whether the STAT3 phosphorylation defect resulted from impaired DOCK8 secondary to the WASP defect, we examined DOCK8 expression in B-LCLs derived from P2, the only patient for whom these cells were available at the time of analysis, and a healthy control. DOCK8 protein levels were comparable between patient and control B-LCLs (**Figure 3E**).

## 4 Discussion

WAS is a rare X-linked IEI first described by Alfred Wiskott in 1937 (22). The clinical spectrum is highly variable, ranging from the milder XLT to the classic severe form, which is characterized by microthrombocytopenia, eczema, recurrent infections and an increased risk of autoimmunity and malignancies (2). Herein, we report three patients harboring LOF mutations in *WAS* gene and exhibiting variable clinicobiological severity. Indeed, P1 presented with an XLT phenotype associated to a mild severity score of 2, whereas P2 and P3 presented with classic WAS, characterized by severe infectious susceptibility and autoimmunity, with scores of 4 and 5, respectively.

Standard immunological investigations revealed eosinophilia in P1 and elevated IgE levels in P2 and P3. Increased eosinophil counts and elevated IgE levels have been frequently observed in WAS patients (23, 24) and may represent key markers to distinguish WAS/XLT from idiopathic thrombocytopenic purpura (3).

Lymphocyte phenotyping revealed a decrease in CD3+ (P2), CD4+ (P2) CD8+ (P2, P3) as well as in non-switched memory B cells (P1, P3) and CXCR5^+^ memory T cells (Tfh cells) (P2, P3). NK cells were increased in all patients. These lymphocyte abnormalities are classically reported in the literature. The reduction in CD4^+^ and CD8^+^ T cells is likely related to defective thymic output, reflecting the essential role of WASp in human lymphocyte maturation (25). The marked decrease in non-switched memory B cells (CD27^+^IgD^+^ B cells) has been described in WAS patients and was attributed to intrinsic developmental defects within the B cell compartment (26). Furthermore, decreased Tfh cell levels have also been reported in WAS patients and WAS-deficient mouse models, correlating with disease severity (27). Interestingly, P2 and P3, both presenting with severe clinical phenotypes, showed reduced levels of Tfh cells. In contrast, these cells were within the normal range in P1, who exhibited a milder clinical presentation, consistent with XLT phenotype. P1 and P3 showed impaired lymphocyte proliferation in response to anti-CD3 stimulation, consistent with the essential function of WASp in early TCR signal transduction (28, 29).

Since the discovery of the *WAS* gene (1), hundreds of pathogenic mutations distributed throughout the entire gene have been continuously reported, significantly advancing our understanding of its genetic basis. Missense mutations are the most frequent and are predominantly located in the first four exons, whereas splice-site variants tend to cluster in exons 6, 8, 9, and 10. In contrast, insertions and deletions are distributed across the entire *WAS* gene (30, 31). A genotype-phenotype correlation has been established in WAS, where the clinical severity reflects the degree of functional WASp protein expression. Missense mutations associated with residual WASp activity usually manifest as XLT, while protein-truncating mutations abolishing WASp expression lead to more severe phenotype (31). However, exceptions to this correlation have been reported. Indeed, several missense mutations can abolish WASp expression yet, still result in milder clinical phenotypes consistent with XLT (30, 32, 33), as observed in P1. The clinical score may evolve with age, warranting regular reassessment and ongoing re-evaluation as part of longitudinal patient monitoring (34). Moreover, P1 carried the p.A56V substitution within the WH1 domain, a critical region for WASp-interacting protein (WIP) chaperone binding that prevents proteasomal degradation (35). This mutation has been previously reported and associated with an XLT phenotype, in line with our clinical observation (30, 33, 36). In those studies, WASp expression was detectable by Western blotting using B-LCLs or PBMCs. In contrast, in our study, WASp expression was evaluated exclusively in T cells by flow cytometry and was undetectable, which may explain the observed discrepancy. In P3, we identified a previously reported splice-site mutation c.734 + 1G>C in intron 7 (37). This variant led to a complete absence of WASp expression, correlating with a severe WAS phenotype (severity score 5). In contrast, P2 carried a previously reported splice-site mutation (c.360 + 1G>T) in intron 3 (38). Residual WASp expression was detected in our patient, suggesting the presence of a functional coding transcript, despite the severe phenotype. Although flow cytometry is routinely used to assess WASp expression in patients, its results may not always perfectly align with genetic findings. Discrepancies occur as mutations in critical domains (e.g., exons 2–4 for WH1/PH or exon 10 for actin polymerization) differentially impact protein stability and function (39). These cases underscore the complexity of genotype-phenotype correlation in WAS and warrants further structural studies to define how different mutants impair protein expression and function.

Given the shared features between WAS and HIES, particularly those caused by STAT3 and DOCK8 deficiencies, we investigated IL-6/STAT3 signaling pathway in T cells of WAS patients to unravel a potential common mechanism. Indeed, WAS and HIES are distinct IEIs that exhibit overlapping phenotype, including recurrent infections, eczema, eosinophilia and elevated IgE levels. Interestingly, despite their clinical and molecular heterogeneity, the three included patients exhibited impaired STAT3 phosphorylation in T cells following IL-6 stimulation. In addition, quantitative real-time PCR analysis of IL-6 stimulated PBMCs from P2 revealed a significant reduction in SOCS3 transcript levels compared to healthy controls. These results, although limited to a small number of WAS-deficient patients, need to be further validated in a larger number of patients as well as on WAS knockout T cell lines. Moreover, the impaired IL-6/STAT3 signaling appears to be independent of DOCK8 expression, as demonstrated in LCLs from P2, which exhibited normal DOCK8 levels. However, this conclusion is based on data from a single patient and limited to protein expression. Further investigations are required to substantiate this hypothesis.

Impaired IL-6/STAT3 signaling in WAS T cells is likely to have broad functional consequences. IL-6 plays a central role in the differentiation of Tfh cells through STAT3-dependent induction of BCL-6, the master regulator of the Tfh program (40, 41). A deficiency in Tfh cells may underlie the impaired humoral immunity and increased susceptibility to infections observed in WAS. Consistent with this, markedly reduced frequencies of circulating Tfh cells were noted in patients P2 and P3, both of whom presented with severe clinical phenotypes. Additionally, IL-6/STAT3 signaling limits early Th2 polarization via induction of SOCS3, which inhibits IL-2/STAT5 signaling (9). Thus, impaired IL-6 signaling may contribute to the Th2-skewed immune profile reported in WAS patients and may be particularly relevant to the pathogenesis of eczema. In this context, dupilumab (anti–IL-4Rα monoclonal antibody), has shown promise as a targeted therapy for managing atopic manifestations in WAS patients (42). Furthermore, IL-6/STAT3 signaling is required for the differentiation of gut-resident RORγt^+^ inducible regulatory T cells (iTregs), which play a critical role in establishing and maintaining oral tolerance (10). This subset deficiency has been associated with food allergy and elevated IgE levels (43). In line with this, WAS patients demonstrate an increased frequency of sensitization to food allergens (44), suggesting a defect in oral tolerance mechanisms. Moreover, WASp-deficient FOXP3^+^ Tregs has been shown to drive Th2-mediated intestinal inflammation and food allergy, marked by increased GATA-3, IL-4, and IgE levels (44). Consistent findings in RORγt-deficient Treg models further support the link between defective mucosal tolerance and Th2-skewed responses to food and commensal antigens (43). Taken together, impaired Il-6/STAT3 signaling in WAS T cells may play a central role in promoting the Th2-biased immune profile, a hallmark shared by WAS, DOCK8, and STAT3 deficiencies. Although likely not the sole driver of immune dysregulation, this defective signaling pathway may significantly contribute to the development of allergic manifestations frequently observed in these disorders.

**Figure 4:**
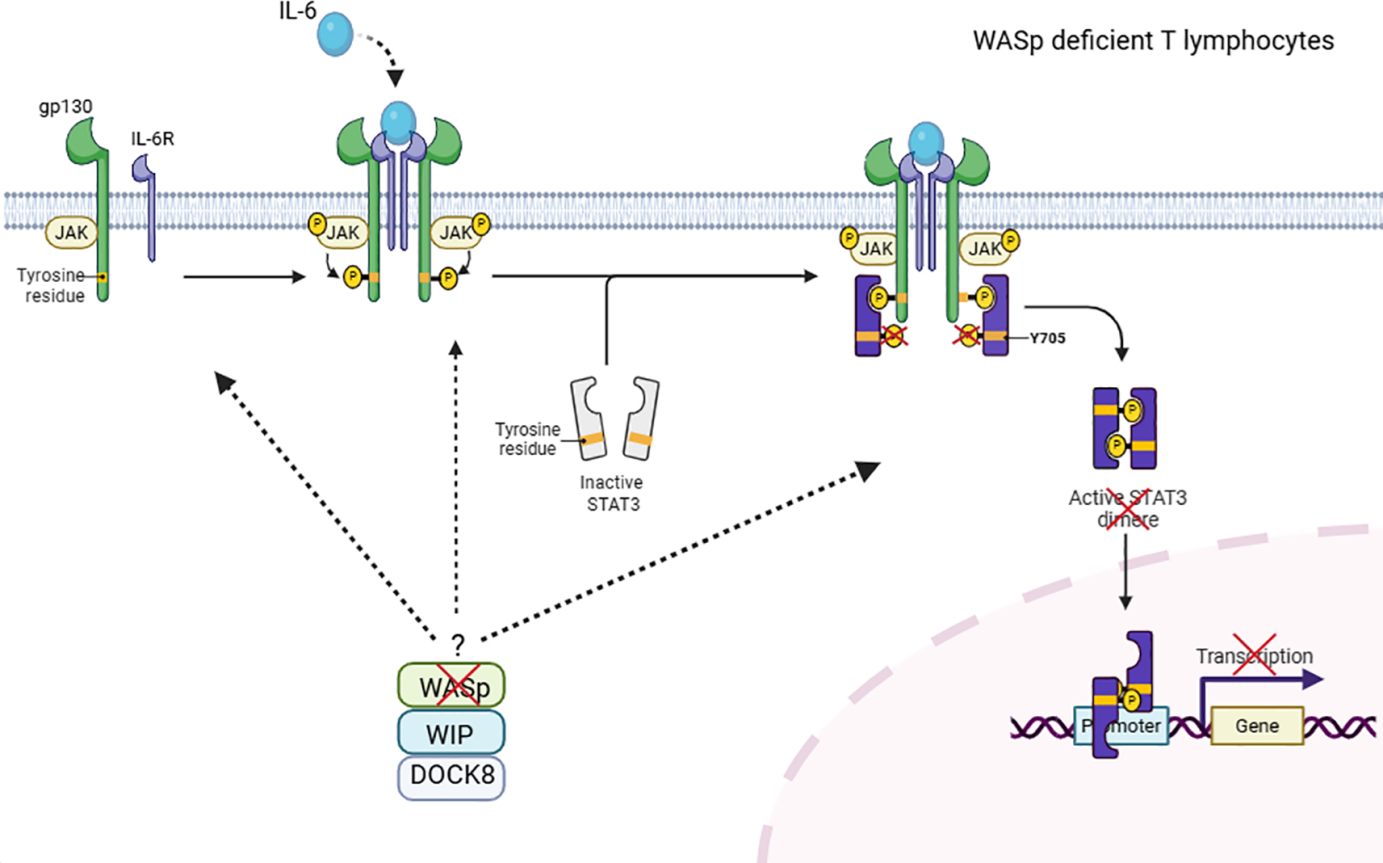
Hypothetical role of the WIP–WASp–DOCK8 complex in IL-6–mediated STAT3 phosphorylation in T cells. DOCK8 has been shown to associate with WASp and WIP to form a molecular complex in T lymphocytes. In this study, we demonstrate that WASp deficiency impairs STAT3-Y705 phosphorylation following IL-6 stimulation in T cells. These findings support a speculative model in which the WIP–WASp–DOCK8 complex contributes to IL-6/STAT3 signaling. The precise mechanism by which the WASP/WIP/DOCK8 complex regulates the IL-6/STAT3 signaling pathway remains to be elucidated. This figure has been created with BioRender.com.

Previous studies have highlighted the role of the WASp-WIP-DOCK8 complex in T cells, where WIP serves as a critical bridge between DOCK8 and WASp in cytoskeletal remodeling (16). Beyond this role, recent evidence suggests that this complex may also contribute to cytokine signaling pathways. In murine macrophages and WAS deficient patients, the WASp–WIP–DOCK8 complex has been shown to regulate IL-10–mediated STAT3 activation (19). In line with this, DOCK8 has been reported to interact constitutively with STAT3 and facilitate its phosphorylation upon IL-6 stimulation via a GEF-dependent mechanism. Indeed, impaired STAT3 phosphorylation has been reported in DOCK8-deficient cells after IL-6 activation (17). Our results extend these findings by demonstrating that WASp-deficient T cells exhibit impaired STAT3 phosphorylation at tyrosine 705 following IL-6 stimulation. Together, these observations support the hypothesis that the WIP–WASp–DOCK8 complex presumably plays a critical role in IL-6/STAT3 signaling in T cells (**Figure 4**). However, how this complex could mechanistically regulate STAT3 phosphorylation in T cells, remains to be elucidated.

In conclusion, our findings add a new piece to the puzzle of the pathophysiology of WAS and provide new insights into the mechanisms underlying its shared features with HIES. The identification of defective IL-6/STAT3 phosphorylation in WASp-deficient T cells highlights the complex interplay among different IEI entities that share common signaling pathways and suggests a potential role for WASp in STAT3 regulation. These results underscore the need for further research to delineate the precise molecular mechanisms by which WASp regulates IL-6/STAT3 activation and to determine whether this regulatory interplay extends to other cytokine signaling pathways. Understanding these interactions could pave the way for therapeutic strategies targeting STAT3 dysfunction in WAS patients.

The **Supplementary Materials** includes **Table 1**, which lists the primer sequences and amplification conditions used for genetic analysis. It also provides detailed protocols for intracellular WASp expression analysis and Western blot analysis of DOCK8 expression.

## Data Availability

The raw data supporting the conclusions of this article will be made available by the authors, without undue reservation.
