# Supplementary material for: Immunogenetic investigation of WAS patients revealing impaired IL-6/STAT3 signaling in T cells
Source: Front Immunol. 2025 Sep 16;16:1602942. doi: 10.3389/fimmu.2025.1602942 (PMC12479476; doi:10.3389/fimmu.2025.1602942)
Supplement: Supplementary file 1 [file DataSheet1.docx]

Supplementary Materials

# Supplementary methods

1. **Western blot analysis of DOCK8 expression**

Western blot for DOCK8 protein expression was performed on Epstein-Barr virus-transformed lymphoblastoid cell lines (LCLs) derived from a WAS patient and a healthy control. Briefly, 5 × 10⁶ cells were collected, washed with an ice-cold solution containing 5mM Tris HCl (pH7.5), 25mM NaCl, 25mM NaF, and then lysed in 50µl of lysing solution. Protein concentration was determined using Bicinchoninic Acid (BCA) Protein Assay (Sigma). Whole cell lysates containing 45µg of proteins were separated by electrophoresis and transferred onto a PVDF membrane. The membranes were incubated in a blocking buffer. They were then probed overnight at 4 °C with either a monoclonal rabbit anti-Human DOCK8 antibody (Abcam, clone EPR1251, 1/5000 dilution) or rabbit anti-β actin antibody (Sigma, anti-actin N-terminal A2103, 1/1000 dilution). After washing, the membranes were incubated with a secondary HRP-conjugated antibody (Sigma, goat anti-rabbit A0545, 1/80,000 dilution). Protein detection was carried out with enhanced chemiluminescence (ECL) substrates (Pierce™ ECL Western Blotting Substrate (Thermo Scientific)).

1. **Intracellular WASp expression analysis**

Heparinized venous blood was collected and Pperipheral blood mononuclear cells (PBMCs) were isolated from heparinized venous bloodo by Ficoll-Hypaque density gradient centrifugation (Eurobio AbCys). Cells PBMCs were aliquoted at 500,000 cells per tube, washed with PBS 0.3% BSA, then fixed and permeabilized for 30 minutes at 4°C using Cytofix/Cytoperm (BD, Cat. No. 554714). After two washes with Perm/Wash solution, cells were incubated with primary antibody solutions (either a 1:200 dilution of mouse anti-human WASp or 1:5 dilution of mouse IgG1 Ab (Becton Dickinson, San Jose, CA)) for 30 minutes at 4°C. Following an additional wash, cells were incubated with a secondary antibody solution (Goat anti-mouse antibody) for 30 minutes in the dark at 4°C and then washed with 2 mL of Perm/Wash. Subsequently, cells were stained with PerCP-Cy5.5-anti-CD3 antibody for 20 minutes, washed with PBS and resuspended in 300 µL of PBS. Samples were then analyzed by flow cytometry.

# Supplementary Table 1

| **Amorces** | **Sequences** | **Exons** | **Size (bp)** | **Tm** |
| --- | --- | --- | --- | --- |
| WAS 1 F | AAGCAGTCAAGTGGAGGAGG | Exon 1 | 400 | 60°C |
| WAS 1 R | GGAAGAGGAAGAAACGGTGG |  |  |  |
| WAS 2 F | GCCTCGCCAGAGAAGACAAG | Exon 2 | 640 | 60°C |
| WAS 2 R | ACTGGCTTGCAAGTCCAGTC |  |  |  |
| WAS 3 F | CTATGAGGCTCCCAAATCCA | Exon 3, 4, 5 and 6 | 790 | 60°C |
| WAS 6 R | ATCCATTCACCCACTTACGC |  |  |  |
| WAS 7F | CTCAAGGCTTCCGTTTCTTG | Exon 7 | 389 | 53°C |
| WAS 7 R | ACCACCCATTTACCCACTCA |  |  |  |
| WAS 8 F | AAGAGGGTTTCACTATGAAGG | Exons 8 and 9 | 796 | 60°C |
| WAS 9R | GCAATCCCCAAAGGTACAGG |  |  |  |
| WAS 10 F | CAGTGGGGGTACCCATTTTA | Exons 10 and 11 | 941 | 60°C |
| WAS 11 R | GGTGACTGCTGGGATTGTTT |  |  |  |
| WAS 12 F | CCTTTCTTGTCCCAAATGGA | Exon 12 | 349 | 60°C |
| WAS 12 R | CAGGCAGGGATAACAGCATT |  |  |  |

F: Forward, R: Reverse, bp: base pair, Tm: melting temperature
